# Public Perceptions of Veterinarians from Social and Online Media Listening

**DOI:** 10.3390/vetsci7020075

**Published:** 2020-06-06

**Authors:** Nicole Widmar, Courtney Bir, John Lai, Christopher Wolf

**Affiliations:** 1 Department of Agricultural Economics, Purdue University, West Lafayette, IN 47907, USA; 2 Department of Agricultural Economics, Oklahoma State University, Stillwater, OK 74078, USA; courtney.bir@okstate.edu; 3 Food and Resource Economics Department, University of Florida, Gainesville, FL 32611, USA; johnlai@ufl.edu; 4 Charles H. Dyson School of Applied Economics and Management, Cornell University, Ithaca, NY 14853, USA; caw364@cornell.edu

**Keywords:** public perceptions, social media analytics, veterinarian, veterinary medicine

## Abstract

The public perception of the veterinary medicine profession is of increasing concern given the mounting challenges facing the industry, ranging from student debt loads to mental health implications arising from compassion fatigue, euthanasia, and other challenging aspects of the profession. This analysis employs social media listening and analysis to discern top themes arising from social and online media posts referencing veterinarians. Social media sentiment analysis is also employed to aid in quantifying the search results, in terms of whether they are positivity/negativity associated. From September 2017-November 2019, over 1.4 million posts and 1.7 million mentions were analyzed; the top domain in the search results was Twitter (74%). The mean net sentiment associated with the search conducted over the time period studied was 52%. The top terms revealed in the searches conducted revolved mainly around care of or concern for pet animals. The recognition of challenges facing the veterinary medicine profession were notably absent, except for the mention of suicide risks. While undeniably influenced by the search terms selected, which were directed towards client–clinic related verbiage, a relative lack of knowledge regarding veterinarians’ roles in human health, food safety/security, and society generally outside of companion animal care was recognized. Future research aimed at determining the value of veterinarians’ contributions to society and, in particular, in the scope of One Health, may aid in forming future communication and education campaigns.

## 1. Introduction

Pet owners can, and do, share information about their pets online. Anyone can easily turn to the Internet for veterinary medicine information, to generate related content themselves, and even share information about the veterinary professionals who care for their own animals (i.e., posting reviews, venting frustrations, or tweeting about office visits). Veterinary practices can obtain the same advantages as many businesses by moving their clinic–client interactions online, including sharing information with clients, increasing interactions with others, increasing accessibility and widening access to health information [1]. Some potential drawbacks include distractions, disagreements, and the low frequency of response [2].

For most pet owners, veterinarians are recognized as caregivers of companion animals and household pets, such as cats and dogs. In the US, 38% of households own a dog, while 25% own a cat; dog and cat owners make, on average, 2.4 and 1.3 veterinary visits per household per year, respectively [3]. Measuring interactions between veterinarians and pet owners can be critical to the success of veterinary medicine practices [4]. Household penetration rates for pet ownership in the U.S. have grown from 62% in 2008 [5] to 67% in 2019 [6]. Internet and social media use has grown from 74% to 90% and 20% to 72%, respectively, in the same time period [7,8]. The transitioning of the World Wide Web towards collaboration and sharing, emphasizing user participation (Web 2.0) affords the veterinary profession another tool for understanding societal wants/needs. These industry trends point toward an increasingly immense amount of publicly available user-generated content and information that can be readily gathered to assess clients’ perceptions. This novel paradigm of veterinarian–client communication is important to the awareness and development of an appropriate and effective relationship [9]. Online and social media analytics regarding veterinary health industries represent a significant divergence from previous methods documented in the literature, which largely revolved around surveys, client reviews/reporting of satisfaction, and client retention rates.

Clients use the collaboration-centric web to gather information and communicate about issues surrounding their companion animals. Veterinarians have also turned to online communication technologies to increase client satisfaction via improved communication, including simple examples like appointment reminders via text or e-mail. Pet owners have been documented as turning to social media in cases of emergency preparedness [10]. Veterinarians can preempt clients to take actions during disaster risks and during other emergencies recognizing the emotional role companion animals play in the resilience of clients [11]. Studies have also found that clients search for health information and share their experiences [12]. Online communication has also been integrated into veterinary program curricula to ensure professional career readiness [13]. Instances of social media use underscore the importance of online communications and represent a significant pathway of information relay for the veterinary profession.

Coupling the literature covering clients’ and veterinarians’ use of social media, it is clear that online social media communication can offer actionable insights for veterinary practices, especially in understanding what the veterinary industry can offer from the perspective of clients [14]. It supports a client-centered approach [15] which is characterized by “time spent building rapport and establishing a partnership with the client, encouraging client questions, inviting clients to share their perspectives, and exploring lifestyle-social topics in the discussion” [16]. The proliferation of the information available on the Internet has provided a guided pathway for conversation between both parties and enables or enhances animal care. However, standardized practices do not exist to assess, nor is there an accepted definition of, an ideal online presence for veterinarians and their practices more broadly.

Guidance on physician–patient relationships is not directly applicable to veterinarian–human client–animal patient relationships due to the nature of the interactions involved. Furthermore, public perceptions of veterinarians and their roles are hypothesized to be unique from other medical professionals given their diverse societal contributions. Limited empirical analysis is available in the veterinary medical literature focused on public perceptions of veterinarians and their various roles in society. There exists a lack of understanding of the role of veterinarians in a One Health environment in which the health of animals, people, and the environment are explicitly intertwined. This analysis seeks to document and summarize the online media chatter related to veterinarians to establish an understanding of what is/is not prominent in the public discussion. The intent of this work is that an understanding of the roles of veterinarians which are most prominent online and through social media may be useful in developing communication approaches and/or to inform training and education to establish an understanding of the roles of veterinary medicine in society more broadly. In the following analysis, researchers first present the materials and methods in Section 2. Next, the results are presented in Section 3, where a firm connection is made to industry-defined roles held by veterinarians in the U.S. Finally, Section 4 provides a discussion and offers valuable pragmatic insights gained for further integration into veterinary practices. 

## 2. Materials and Methods 

A number of database and online/web search tools are available, some of which are tailor-made for specific searches, such as for searching case law or legal documents, whereas others are broader or news media focused. For example, LexisNexis provides universities and government agencies with news and business sources and searching capabilities (LexisNexis, 2018). Recent advances in online media, and in particular Web 2.0 technology which facilitates posting/sharing by users, have led to an interest in searching social and online media. Increasingly, social media is a platform, available in multiple languages and inviting voluntary contributions from users worldwide that provides searchable databases of the responses, comments, and sentiments of individuals.

### 2.1. Social Media Listening Approach

The Netbase platform [17] was employed to study the number of online posts, from Twitter and other publicly available sites including blogs, news releases, and online publications, related to keywords associated with veterinarians over the time period from 1st September 2017 to 30th November 2019. The Netbase platform is a recognized leader in social media search engines, listening, analytics, and social media intelligence. The time period over which data was collected offers over 2 years of data, facilitating inclusion of both positive and negative news-worthy events and in-depth study of a time period long enough to allow for impacts due to seasonality or other annual factors. Due to the nature of social media and online data searches in which accounts, posters, or individual media/posts can be removed or reinstated by the author or the social media platform itself, the specific date of data collection is imperative to note. Data for the time period of 1st September 2017 through 30th November 2019 was collected on 10th December 2019.

Although searches in multiple languages are technologically possible, the language interpretation, slang vernacular used, and shorthand of online media posts must be acknowledged and considered by researchers, and thus this analysis was limited to posts in English. For many topics, but especially within the links between animal species, human caregivers, and pets, there are cultural norms and expectations that vary by geographic region and country. Cultural context makes country-specific analysis valuable to facilitate the interpretation of findings. While social listening is technically possible across multiple countries, for this analysis, the geography for all searches employed was limited to the United States, the U.S. Virgin Islands, and Puerto Rico. 

### 2.2. Search Term Development and Implementation

Primary search terms, or keywords, along with exclusionary terms, were developed by researchers to facilitate the collection of a dataset encompassing online and social media posts associated with veterinarians, veterinary medicine, and veterinary service locations (i.e., animal hospitals or clinics). Primary search terms employed in this search and analysis were veterinarian, #veterinarian, vet office, vet clinic, animal clinic, animal hospital, cat hospital, pet clinic, veterinary medicine, pet hospital. Exclusionary terms were identified by researchers through the process of ‘tuning’ the search, by scanning search hits to identify extraneous media hits and developing exclusionary terms to address top extraneous findings. Exclusionary terms used in this analysis were burned body of a woman, woman’s burned corpse, Hyderabad, #bookclub, #nj, #book, #asmg, #kindle, #mystery, Megan Stanford, @HallieTurich, have been lunged, 31/2 years. Two domains were excluded, as they were identified as returning erroneous search hits, specifically boards.4chan.org and yellowbot.com. Two authors were excluded from the search and analysis conducted, namely indopremier.com:flickr.com and MainChannel_:twitter.com. 

### 2.3. Social Media Sentiment Analysis and Reporting 

Sentiment, generally speaking, is the opinion, view, or attitude towards a situation, event, topic, or occurrence. Net sentiment, as it is commonly referred to within the social media listening framework and the literature, is fundamentally a construct of comparing positive and negative posts to arrive at a single value that captures the positivity/negativity of the posts returned in each search/analysis. A third category of posts, neutral, is also employed when calculating and reporting on analytics for top words and other data summaries, but is not used in the calculation of net sentiment. The net sentiment referenced throughout this analysis is the result of the total percentage of positive posts minus the percentage of negative posts, thus resulting in a net sentiment that is necessarily bounded between −100% and +100%. Social media sentiment was analyzed using the Natural Language Processing (NLP) capabilities of the Netbase platform [18,19].

### 2.4. Comparing Social Media Top Terms to Publicly Recognized Roles of U.S. Veterinarians 

The American Veterinary Medicine Association (AVMA) serves as a leading source of information for both the veterinary medical profession and the general public via information accessible on their website and associated media about the profession. The AVMA offers information for pet owners about their veterinarian; pet owners visiting the AVMA website who select resources and tools, and then to learn about their veterinarian, face a list of available references (https://www.avma.org/resources-tools/pet-owners/yourvet) including “finding a veterinarian” and “8 things to consider when choosing a veterinarian”. There is also a link on the AVMA main page to a webpage titled “Veterinarians: Protecting the Health of Animals and People” [20] (AVMA, 2019 https://www.avma.org/resources/pet-owners/yourvet/veterinarians-protecting-health-animals-and-people) which was employed in this analysis to serve as a holistic list of the roles of veterinarians in U.S. society. Sixteen total roles were identified and summarized in Table 1 by employing the AVMA (2019) resource to serve as a point of comparison to the search results yielded from the online media search. 

## 3. Results

Over 1.7 million mentions were captured from over 1.4 million posts from the September 2017 to November 2019 time period evaluated. The number of mentions, average sentiment, timing of posts (day of the week) and top domains and sources for search results are displayed in Table 2. Day of the week posts are available from any source for which a time stamp was available. In total, the day of the week can be ascertained for 139,870 data points in the search conducted. The day of the week for posts was slightly higher early in the work week (Monday, Tuesday) and lower on the weekends (Saturday, Sunday), but otherwise showed no large deviations in most/least popular days of the week. The top domain for search results was Twitter (74%), although reddit.com (9%) and Instagram.com (7%) both yielded measurable contributions to search results.

Net sentiment was evaluated from 163,597 posts for which positive or negative sentiment could be ascertained, which was 9% of the total mentions captured by the search parameterized. In total, 76% of the mentions with sentiment were positive and 24% were negative, yielding a net sentiment of 52%. 

The inferred demographics of posters on Twitter are displayed in Table 3, offering insight into who is posting and what interests those posters have, broadly speaking. Admittedly, ascertaining biographical or demographic information of posters from online media is complicated. Gender was obtained either from an author/poster statement in their own biographical information or assumed using common baby naming databases (Netbase, 2018). Using the 398,354 posts for which gender could be inferred, this search yielded posts by more females (57%) than males (43%). Estimated ages are available for sources that include a first name and used the methods employed by the Netbase platform, which impose estimated ages based on names and Social Security Administration name data. “Age classification is based on the popularity of first names by year of birth according to U.S. Social Security Administration data, which makes this feature more relevant for U.S. markets. This data includes about 65,000 of the most popular first names, covering more than 80% of the U.S. population” (Netbase, 2018). In total, 46% of the posts for which inferred age was assigned (*n* = 398,525) were from posters 45 years of age or older. Generally, this search did not reveal large numbers of posts coming from a specific age bracket of posters. Interests were determined using keywords in the biography text written by Twitter users. Unsurprisingly, pets were tied as the top two interest of posters in the search conducted about veterinary medicine. The top two interests revealed from the 185,543 posters for which interests were discernable were family (28%) and pets (27%), with a very large differential with the third top interest, politics, at only 12%. 

**Figure 1:**
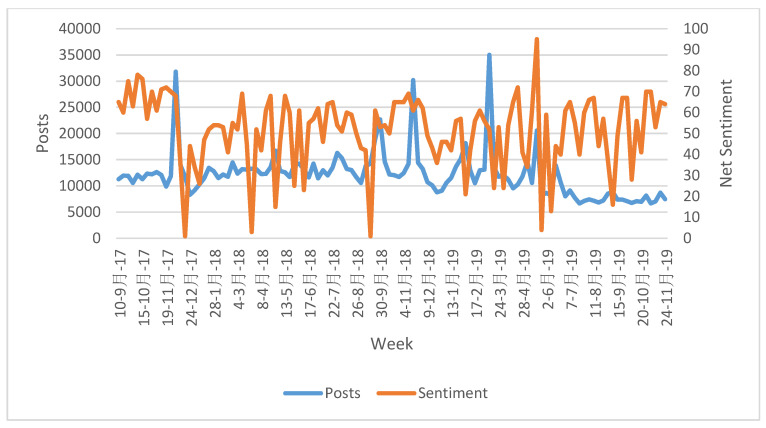
Weekly posts and net sentiment from September 2017 through November 2019.

Figure 1 displays the weekly number of posts and weekly net sentiment for the search conducted. Evaluating weekly numbers of mentions and posts from the September 2017 to November 2019 time period reveals that weekly posts ranged from a low of 6,653 in July 2019 to a high of 35,008 in March of 2018. The standard deviation among weekly posts was 4,424. Sentiment ranged from a low of 1 in December of 2017 and September of 2018 to a high of 95 in May of 2019. Recall that net sentiment ranges from a possible low of −100 and high of +100; thus, even at the lowest recorded weekly net sentiments, the search results remained positive. The average net sentiment over the whole time period studied was 52%, with a standard deviation among the weekly measurements of 17 percentage points.

The week of 17th December 2017 saw a significant dip in net sentiment to 1%, dropping from 72% and 70% just 3–4 weeks earlier. Looking at the week of December 17th specifically, 30% of the net sentiment was driven by online media mentioning caring for a new puppy and being on watch for diarrhea, and 22% was driven by media-fueled chatter about a veterinarian in Louisiana shooting a neighbor’s dog in the head. Moreover, the drop in total mentions in that same week is also notable, having dropped from over 31,816 in the week of December 3rd, with a 68% net sentiment, to only 11,734 in the week of 17th December 2017. The week of 25th March 2018 saw net sentiment drop to 3% in another downward spike, with drivers being mentions of the dangers of medicating dogs on flights, commentary about a skin condition in giraffes being investigated via questionnaires with veterinarians, and reporting on a pit bull which was abused in August 2013, but was subsequently re-publicized in news media about abuse. Sentiment in the week of 16th September 2018 was influenced most heavily (23%) by stories related to a woman who was charged with practicing veterinary medicine without a license after taking in animals effected by Hurricane Florence. Other headlines included adverse reactions to medications and possible health and safety issues related to overheating while being dried during grooming if precautions were not employed. A significant positive spike in sentiment was apparent in the week of 19th May 2019, driven largely by two references. The first was to a family member of the founding Prime Minister of Singapore with a photo of dogs balancing wedding bands and the mention of employment as a veterinarian. The second to the Standardbred Retirement Foundation having an online auction fundraising event that included the opportunity to shadow a small animal veterinarian.

Aside from movements in net sentiment or numbers of mentions, the specific attributes, behaviors, terms, and hashtags which drove positive or negative sentiment are displayed in Table 4. Top positive attributes referenced treating dogs (16%) and comfort dog assistant (14%), with dog again tied as the top positive term. Positive sentiment within hashtags was driven largely by career-oriented words such as #hiring, #job, and #careerarc. Interestingly, the only corporation/corporate veterinary clinic to organically appear as a top search result, under things, was Banfield (7%).

### Comparing Search Results to Roles of Veterinarians as Identified from AVMA Resources

Roles for U.S. veterinarians range from caring for animals directly, in animal shelters, pets kept in client’s homes, and zoos/aquariums to working with the Centers for Disease Control and Prevention (CDC) and National Institutes of Health (NIH) on issues pertinent to both human and animal health and well-being through One Health initiatives. Table 1 summarizes the roles of U.S. veterinarians as identified from AVMA [21]. Sixteen total roles of veterinarians in the U.S. were documented, although the roles beyond direct pet animal care did not arise organically as top mentions or terms in the searches conducted. Given the search intent focused on veterinarians in the client-facing realm of pet hospitals and animal clinics, the lack of diversity of the roles mentioned may be expected. Nonetheless, the lack of mention of any of the roles of veterinarians influencing human wellbeing may point to room for further promotion of the diverse roles of veterinarians. 

## 4. Discussion

Online media analytics have been used in a variety of industries to study topics ranging from responses to natural disasters [22], to investigating product development [23], to understanding social media in tourism industries [24]. In particular, the advent of social media in which many users create and share content via Web 2.0, as opposed to a few content creators broadcasting to many readers/consumers, has changed how online media is used in terms of ongoing conversations, rather than simple posting of news or media pieces [25]. Broad topics of conversation incorporating veterinarians and veterinary medicine were uncovered in the search conducted, with over 1.7 million total mentions captured. While no benchmark for mentions exists, as searches are individually parameterized, the overall quantity of search hits was sufficient to see movement over time and to capture top terms of interest arising in the search results. Given the topic, veterinarians and veterinary medicine, there was not a clear pattern expected with respect to the days of the week for posts. Given the common business hours of many veterinary clinics and pet hospitals, the slightly higher numbers of posts early in the work week with lower numbers of posts on the weekends were reasonable, although no known/verified point of comparison was available given the nature of the data collection and analysis. 

While the inferred demographics of posters from Twitter data can offer some insight into who is posting, ascertaining known demographics in online and social media is complicated and wrought with biases associated with self-reporting. Using the inferred gender of posters, more females than males were posting media that was captured in the searches provided. Bir et al., in a study about dog acquisition, reported the demographics of pet owning households and non-pet owning households side-by-side from a nationally representative sample [26]. Bir et al. found that owning a pet was more commonly reported by females, by larger households, and by respondents under the age of 65 [26]. While not directly related to pet ownership, McKendree et al. reported on the demographics of those most concerned about animal welfare, revealing that females were more likely to self-report concern [27]. Taken together, the overrepresentation in search results about veterinarians and veterinary medicine by females is in keeping with suggestions by previous literature that females may more often report pet ownership/care duties and concern for animals, broadly speaking. Ages of posters are complicated to interpret, as social media usage may drive some of the lower participation by older demographics. However, pet ownership has been documented most commonly in middle-aged households and less so in households with residents over 65 years of age [26], so it is likely that both social media user demographics and pet owner demographics were contributing to posts across the age brackets reported in this study. 

Net sentiment can range from −100% to +100%, making the net sentiment of 52% a clearly positive overall assessment as captured in the results over the time period in question. Notable events impacting sentiment tended to be about animals themselves, such as harm to animals or risking animal’s wellbeing during travel. Career-oriented terms were largely within the hashtags, such as #hiring, which is also undeniably related to the domains in which online media appears (i.e., Twitter). In a very small number of posts, at 1%, a top negative attribute was die by suicide, which is an aspect of the profession that has received significant attention in the media and press [28]. Risk factors among veterinarians, such as suicidal ideation, attempts at one’s own life, and depression, have been cited in the literature, both in the U.S. and in other countries [29,30,31,32,33]. Other challenges facing the veterinary health profession are well documented within the industry, including student debt loads [34], mental health implications arising from compassion fatigue [35], and the impact of performing euthanasia [36], in addition to lesser discussed but more obvious risks to (human) physical safety arising from working with animals [37]. While these challenges and issues are being raised in the media and in the literature around the world, veterinary suicides were not found in the searches conducted in this study.

There was a general lack of top terms revealed for the veterinarian searches conducted that pertained to roles outside of caring for household pets and companion animals. Roles identified for U.S. veterinarians by the AVMA ranged from caring for animals directly, in animal shelters, pets kept in client’s homes, and zoos/aquariums to working with the Centers for Disease Control and Prevention (CDC) and National Institutes of Health (NIH) on issues pertinent to both human and animal health and wellbeing through One Health initiatives [21].

## 5. Conclusions

Online media, including user-generated posts such as those on social media platforms, is increasingly popular as a source of information and communication among consumers and members of the general public. In this analysis, the issues facing the veterinary medicine profession from within were, perhaps unsurprisingly, largely absent from the search results, which focused heavily on the pet owner or consumer/client side of veterinary medicine. Search terms reveal a focus on pet animal welfare, illnesses, and care, with some hashtags pointing towards career or profession advancement. Notably absent in the search results were references to roles embodying One Health initiatives in which the links between the health of animals, humans, and the environment are explicit.

Undoubtedly the search terms selected directed searches to capture verbiage from veterinary clients, thus driving results towards the companion/pet animal aspects of veterinary medicine. However, within those results were career promotion hashtags and the recognition of suicide risks. It is possible that roles beyond client–animal–clinic relationships are simply relatively less discussed in online media, or that the searches conducted did not adequately target societal roles outside of direct pet caretaking. Surely, the popularity of discussing pets in social media space contributes to the abundance of mentions related to direct pet animal caretaking. It is impossible to know if career promotion, mental health risks, and/or any of the other comments originated from outside or within the profession. In other words, it could be that social media posts from veterinary medical professionals are referencing these topics.

While ongoing concerns and public health campaigns about tick-borne illnesses and rabies continued (and a focus on other higher profile risks like Ebola emerged) during the study time period, the public-facing focus on public health was far less relevant than it is today in light of an ongoing coronavirus (COVID-19) pandemic situation. Perhaps the increased focus on zoonotic diseases in recent months and, in particular, in popular news media, may increase the attention and recognition of One Health and the roles of veterinarians in public health. Regardless of the degree to which search terms and/or the timing of data collection contributed to these findings, the relative lack of any mention of the roles of veterinarians in One Health endeavors may prompt further research into the perceptions of veterinarians in the public domain [38]. In particular, future work may wish to broaden searches to determine if discussions of human medical needs may more directly reference veterinarian contributions [39,40,41]. Nonetheless, veterinary health organizations and associations may consider the broader dissemination, when appropriate, of information about the diverse roles that veterinarians play in society. 

## Figures and Tables

**Table 1:** Roles of veterinarians in the U.S. identified from American Veterinary Medicine Association (AVMA) publicly available resources.

Care for zoo/wildlife/non-domestic animals and aquatic animals and fish residing in aquariums, wildlife sanctuaries, and other parks
Care for pet animals
Work in Centers for Disease Control and Prevention (CDC) to protect human populations from disease
Work in animal shelters and rescue operations
Work in USDA Animal and Plant Health Inspection Service (APHIS) to monitor the development of vaccines for safety and effectiveness
Care for farm animals, including those entering the food system (e.g., cows, pigs, chickens, turkeys, sheep, etc.)
Work in the National Institutes of Health (NIH) and its National Library of Medicine
Work in the US Air Force Biomedical Science Corps as public health officers
Work in private industries providing animal care and conducting research in animal and human health
Work in US Army Veterinary Corps providing protection from bioterrorism and care of government-owned animals and their interests
Work in USDA Agricultural Research Service (ARS) and the National Institute of Food and Agriculture (NIFA) for research, research administration, and animal care
Work in the Environmental Protection Agency (EPA) studying the effects of pesticides, industrial pollutants, and other contaminants on animals and humans
Work in the US Food and Drug Administration (FDA), evaluating the safety and efficacy of medicines, medical products, pet foods, and food additives
Work in the USDA Food Safety and Inspection Service (FSIS) to ensure safety of the human food supply
Work in the US Department of Homeland Security developing disease surveillance and antiterrorism procedures and protocols
Work in USDA Animal and Plant Health Inspection Service (APHIS) for disease surveillance to prevent foreign animal diseases from entering the country

**Table 2:** Mentions, average sentiment, day of week and top 10 domains and sources.

**Mentions (n)**	1,799,066
**All Posts (n)**	1,405,899
**Net Sentiment (n)**	163,597
**Mean Net Sentiment September 2017–2019**	52%
**Posts by Day of Week (n)**	139,870
**Monday**	17%
**Tuesday**	18%
**Wednesday**	15%
**Thursday**	15%
**Friday**	15%
**Saturday**	11%
**Sunday**	10%
**Top 10 Domains (n)**	946,983
**twitter.com**	74%
**reddit.com**	9%
**instagram.com**	7%
**justanswer.com**	4%
**ihs.jobs**	1%
**forums.studentdoctor.net**	1%
**veterinarypracticenews.com**	1%
**thehorse.com**	1%
**my.jobs**	1%
**dogsnow.com**	1%
**Top 10 sources (n)**	1,798,953
**Twitter**	38%
**News**	36%
**Forums**	12%
**Blogs**	11%
**Instagram**	4%
**Consumer Reviews**	<1%
**Comments**	<1%
**Professional Reviews**	<1%
**Other Social Networks**	<1%
**Facebook**	<1%

**Table 3:** Inferred demographic information of Twitter posts.

Demographics	Percentage of Posters
Gender (*n* = 398,354)	
Male	43%
Female	57%
Age (*n* = 398,525)	
<18	9%
18–24	12%
25–34	17%
35–44	15%
45–54	15%
55–64	19%
65+	12%
Top 6 interests (*n* = 185,543)	
Family	28%
Pets	27%
Politics	12%
Music	11%
Religion	10%
Food and Drink	9%

**Table 4:** Top five positive and negative sentiment drivers for different word categories.

Positive		Negative	
Attributes (*n =* 89,309)			
treat dog	16%	Animal	2%
comfort dog assistant	14%	die by suicide	1%
help	7%	die	1%
work	4%	issue	1%
offer to board pet	4%	kill	1%
care	3%	shoot dog	1%
Behavior (*n* = 19,432)			
work	16%	accuse	7%
want	15%	charge	5%
recommend	9%	not go	1%
look for	9%	prohibit	1%
support	5%	sue	1%
use	5%	arrest	1%
helps	11%	dog	3%
dog	11%	warn	1%
alright	6%	owners	1%
comfort dog assistant	6%	accused	1%
sick dog patients	6%	died	1%
work	5%	puppies	1%
#hiring	16%	#vaccines	<1%
#job	15%	#dog	<1%
#jobs	9%	#dogsofinstagram	<1%
#veterinary	8%	#alj	<1%
#careerarc	7%	#federalinsider	<1%
#assistant	4%	#dogs	<1%
Things (*n* = 119,150) Collected mentions without positive/negative association			
hospital		22%	
animal		18%	
dog		11%	
comfort dog assistant		10%	
Vet		8%	
Banfield		7%	
